# Nanoparticle exposure at nanotechnology workplaces: A review

**DOI:** 10.1186/1743-8977-8-22

**Published:** 2011-07-27

**Authors:** Thomas AJ Kuhlbusch, Christof Asbach, Heinz Fissan, Daniel Göhler, Michael Stintz

**Affiliations:** 1 Air Quality & Sustainable Nanotechnology, Institute of Energy and Environmental Technology e.V. (IUTA), D-47229 Duisburg, Germany; 2 Research Group Mechanical Process Engineering, Institute of Process Engineering and Environmental Technology, Faculty of Mechanical Engineering, Technische Universität Dresden (TUD), D-01062 Dresden, Germany; 3 Center for Nanointegration Duisburg-Essen (CeNIDE), University Duisburg-Essen, D-47057, Germany

**Keywords:** Nanoobjects, nanomaterial, airborne, release, exposure, workplace, handling, processing

## Abstract

Risk, associated with nanomaterial use, is determined by exposure and hazard potential of these materials. Both topics cannot be evaluated absolutely independently. Realistic dose concentrations should be tested based on stringent exposure assessments for the corresponding nanomaterial taking into account also the environmental and product matrix. This review focuses on current available information from peer reviewed publications related to airborne nanomaterial exposure. Two approaches to derive realistic exposure values are differentiated and independently presented; those based on workplace measurements and the others based on simulations in laboratories. An assessment of the current available workplace measurement data using a matrix, which is related to nanomaterials and work processes, shows, that data are available on the likelihood of release and possible exposure. Laboratory studies are seen as an important complementary source of information on particle release processes and hence for possible exposure. In both cases, whether workplace measurements or laboratories studies, the issue of background particles is a major problem. From this review, major areas for future activities and focal points are identified.

## 1. Introduction

Research and product developments in the area of nanotechnology have steadily increased especially due to new, beneficial properties of nanomaterials. Nanotechnology as a cross-cutting technology, nowadays used in electrical devices, in construction and composite materials, as catalysts and as antibacterial coatings, is more and more present in workplaces as well as consumer products. This steady increase is accompanied with larger production, handling and processing facilities for nanostructured materials and higher tonnage of nanomaterials.

New nanomaterials, an inherent part of nanotechnological developments, allow on the one hand new products and solutions to e.g. societal problems related to natural resources, drinking water, energy generation and storage, but also raise concerns due to their new specific properties. The major concern is, that the new properties and the high mobility of some nanomaterials may lead to health or environmental effects. This concern has been identified early and was taken seriously by public bodies and the industry. First specific research investigations in toxicology related to particles at the nanoscale were already conducted in the late 1980's [1]. During the last two decades the amount of toxicological research on nanomaterials has increased from less than 10 publications before 1998 to more than 200 in 2010 (ISI Web of Knowledge, 02/2011). A risk, however, may only arise if both a hazard potential of the nanomaterial and exposure exist. Therefore first studies of workplace related exposure were initiated by the International Carbon Black Association (ICBA) in 1998 [2,3]. In parallel Maynard et al. [4] conducted first studies related to nanomaterial exposure of carbon nanotubes (CNT). The number of workplace studies and published results has increased significantly since then and a first ISO-guideline on inhalation exposure characterization and assessment has been set-up [5].

Different approaches can be pursued to derive exposure relevant information in workplaces: (a) Studies based on real workplaces and (b) process based studies in simulated workplaces and of simulated work processes. The major advantage of the prior approach is that data from real work conditions are obtained. Still, due to various background aerosols originating from the general work environment or the process itself, extensive and time consuming measurement campaigns have to be conducted. The latter approach, based on simulations in laboratories allows the clear differentiation of a release from the investigated process from background aerosols coming from other sources than the process. It has to be noted that background aerosols may stem from areas outside of the work area and from the process itself. The latter can again be divided into background aerosols released from e.g. electrical motors used in the process [6-8] or unintentionally produced particles from the production process as a side product. This clear differentiation of background aerosols is also necessary to understand what type of background aerosol can be differentiated by which measurement approach.

Process based studies in simulated workplaces and of simulated work processes also enable investigations on how variances in handling, process conditions influence release rates. Release rates are important pieces of information for workplace modelling approaches as investigated and described in detail by Schneider et. al. [9]. Still, laboratory simulations do not represent the real work conditions but represent an important complementary way to derive information on possible nanomaterial exposure at workplaces.

The purpose of this manuscript is to present an overview of publications related to the exposure of engineered nanomaterials in the workplace and processes which may lead to such exposure. Workplace investigations and laboratory simulations are presented and discussed separately since strategies and methodologies employed differ significantly for both approaches. In some cases references to investigations are given in both parts. Those are valuable studies linking process studies with real work conditions, even though this quite often only refers to small bench workplaces.

## 2. Methods, devices and measurement strategies for airborne nanoobjects and nanomaterials

### Methods and devices

Measurement and sampling devices for airborne nanoscale particles used in workplace exposure studies can generally be divided into four types, i.e. any combination of size resolved and size integrated with time resolved and time integrated as shown in Table 1.

**Table 1:** Selected measurement and sampling devices for airborne particles

Measurement/sampling device	Size range/time resolution/metric + equivalent diameter	References
**Size resolved**		
*Time resolved*		

Scanning mobility particle sizer (SMPS)	2.5 nm - 1000 nm > 30 s number size distribution, based on electrical mobility diameter	[10,11]

Electrometer based mobility particle sizer: fast mobility particle sizer (FMPS)/engine exhaust particle sizer (EEPS)	5.6 nm - 560 nm 1 s/0.1 s number size distribution, based on electrical mobility diameter	[12,13]

Electrical low pressure impactor (ELPI)	6 nm - 10 μm 0.1 s number size distribution, based on aerodynamic dia.	[14]

Optical particle sizer (OPS):- laser aerosol spectrometer (LAS)	(> 60 nm) > 300 nm - 20 μm 1 s number size distribution, based on light scattering equivalent dia.	[15]

Inertial spectrometer/time of flight instruments: - aerodynamic particle sizer (APS)	500 nm - 20 μm 1 s number size distribution of the aerodynamic dia.	[16]

**Size resolved**		
*Time integrated*		

Low pressure cascade impactor	> 20 nm n.a. mass size distribution, chemical analysis, morphology	[17,18]

Micro orifice uniform deposit impactor (Moudi)	10 nm -20 μm n.a. mass size distribution, chemical analysis, morphology	[18]

Wide range aerosol system (WRAS)	5.5 nm - 32 μm 5 min number size distribution	[19]

Thermal precipitator (TP)	20 - ca. 300 nm n.a. size distribution	[20]

**Size integrated**		
*Time resolved*		

Condensation particle counter (CPC)	5.5 nm - 9 μm 1 s particle number concentration (NC)	[21,22]

Surface area monitors (e.g. electrical aerosol detector (EAD), nanoparticle surface area monitor (NSAM), LQ1-DC)	10 nm - > 1 μm 1 s aerosol length (EAD), active surface area (LQ1-DC), lung deposited surface area (NSAM)	[23-25]

Aerosol photometer	250 nm - 20 μm 1 s mass concentration	

**Size integrated**		
*Time integrated*		

Electrostatic precipitator (ESP)	> 20 nm n.a. chemical analysis, morphology	[26]

Thermal precipitator (TP)	> 20 nm n.a. chemical analysis, morphology	[20]

Filtration (e.g. PM10, PM2.5)	mass concentration, chemical composition	

#### Size resolved, time resolved

These devices usually use detection principles based on the particles' optical properties or electrical mobility. Hence the results are based on optically or electrically equivalent spheres. In electrical mobility analysis, the equivalence is only valid for the given particle orientation in the classifier, which in case of non-spherical particles may be unknown and hence bias the measurement accuracy. Handling and calibration of mobility particle sizers are standardised in ISO 15900:2009 [27]. Problems of equivalency for different particles also exist for optical detection methods if optical properties vary significantly, e.g. carbon black versus TiO_2_. It also is important to take the time resolution into account. Particle size distributions may vary in time scales of seconds which is not resolved by the SMPS (3-5 minutes time resolution) but often used for such measurements. Hence fast particle sizers like FMPS or ELPI should be employed for example to analyse fast changes in the particle size distributions.

#### Size integrated, time resolved

The particle size weighting is dependent on the metric. Number based detectors give the same signal weight to each particle, while geometric surface area based devices increase the signal weight according to the particle diameter squared. Condensation particle counters (CPC) detect particles from a few nanometres to a few micrometres [22], depending on the model used. Contribution of particles to the surface area is often below detection limit at particle sizes below approximately 20 nm due to the metric immanent weighting of particle size distributions towards larger particles. Diffusion charger based surface area monitors are most accurate in a size range from approximately 20 nm to 400 nm [28]. The lower detection limit is usually not critical due to the low surface area contribution of such small particles in most real particle size distributions. The upper limit, however, may cause more significant errors, because even a small number of these large particles can have a significant contribution to the total surface area.

#### Size resolved, time integrated

Stahlmecke et al. [29] showed that particles tend to further deagglomerate with increasing pressure difference across an orifice. Hence devices like the low pressure impactor may lead to significantly artificial change of the particle size distribution in the measuring device. A second major error using time integration is the change in physical and chemical characteristics during sampling on the substrate due to interaction with other particles or the gas. One example is the adsorption of volatile organic gases onto collected particles and the substrate.

#### Size integrated, time integrated

The ESP or TP as shown in Table 1 can efficiently sample down to 20 nm and below. While both types of samplers can generally also sample micrometre sized particles, they can easily be lost in the sampling lines or inlet system. Thermophoretic deposition is more or less independent of particle size up to approximately 400 nm. Hence TPs can provide a homogenous deposit of particles which can also be evaluated for the particle size distribution [20]. The sampling efficiency for fibrous particles in TPs has to be investigated since inefficiencies in collection may exist.

Common measurement methods of time integrated samples applied after collection are the bulk chemical analysis of a filtration or cascade impactor sample, morphological studies using electron microscopy (SEM, TEM) and single particle chemical analysis using Energy Dispersive X-ray analysis (EDX).

More detailed information on measurement techniques for airborne nanoscale particles are given in [15,30-32].

Despite all the progress made during the recent years with regard to measurement techniques and strategies for exposure assessments towards airborne nanomaterial, there is still the lack of clear performance criteria to achieve comparability and hence nanomaterial specific workplace safety criteria. The development of easy to use devices for the measurements by hygienists is urgently needed even though no agreement on the best metric exists. First personal samplers for time resolved nanoparticle measurements have been recently developed. Such devices can currently not be employed in the breathing zones of workers. In summary, we currently lack information on personal exposure and rely on estimates based on static measurements and first measurements with recently developed devices.

### Measurement strategies

Measurement techniques and measurement strategies have to be optimally combined to allow sensitive and cost effective determination of airborne engineered nanomaterials at workplaces. Measurement strategies can mean tiered approaches as has been suggested by [33] and [34]. Tiered approaches may facilitate cost effective screening of many workplaces if sensitive sensors exists. The NEAT strategy, as developed by NIOSH [33], applies a handheld device which determines particle number concentrations. Particle number concentrations are quite sensitive for nanoscale particles and thus a real-time screening allows identification of possible "hot spots". If the initial screening reveals that the workplace is "clean", i.e. measured concentrations are below a certain threshold value, no further investigations may be necessary. If a nanomaterial release is suspected due to increased concentration levels, the next step of investigations of the tiered approach will be more detailed. This can be personal exposure related, process related or approaches closely linked to toxicological and epidemiological questions.

Directly after the screening detailed measurements related to the above mentioned aims and approaches may be pursued in the areas, which are possibly affected by airborne nanomaterial. Personal exposure approaches will either be based on personal devices and samplers or areal measurements combined with the recording of personal activity patterns to allow the calculation of personal exposure. So far no legal binding framework concerning nanoparticle specific limit values exists. Personal exposure studies related to nanomaterial exposure are therefore dominantly linked to research studies. A good discussion can be found in Maynard and Aitken [35].

Process related approaches will mainly be based on real measurements, allowing for the concurrent use of several and more sensitive devices. Measurement locations will be often close to the process, handling or work activity of interest.

Approaches with the aim of deriving detailed information for toxicological and/or epidemiological studies will use a variety of sampling and measurement devices with possibly health relevant metrics. The variety of devices used for this purpose is high as the metric which best fits to nanomaterial related health effects is still under discussion.

One considerable problem is the distinction of nanomaterial from the background aerosol. Four basically different approaches for background distinction can be differentiated as followed:

▪ time series approach,

▪ spatial approach,

▪ approach based on comparative studies with and without nanomaterial,

▪ (size resolved) chemical and/or morphological analysis.

The different background distinction approaches are part of the measurement strategies presented above. The task to be tackled determines the combination of needed measurement strategy and devices. Time series analysis is generally coupled with online detection methods. This analysis basically assumes that the concentration determined during no work activity is the background concentration and any increased concentrations during the work activity can be attributed to the process, the nanomaterial or both.

The spatial analysis assumes that a background measurement location is representative for the background at the workplace of interest. Any difference between the determined background and workplace concentrations can be linked to the work activity and the nanomaterial investigated.

Most of the studies published so far use a combined approach of time series and spatial analysis, or link the spatial analysis with morphological or chemical analysis (see Table 2). Morphological analyses are often included when single particle chemical analyses are conducted (SEM-EDX) or distinct features of the nanomaterial can be used for its identification like a fibre structure.

**Table 2:** Measurement Strategies for background distinction (only articles with original measurements)

	With/without activity	With/without nanomaterial
Time series analysis	[36-41]	[42]^2,3^

Time series and, or only spatial analysis	[2]^2^, [3]^2^, [33]^2,3^, [36]^1^, [38]^1^, [43-52]^2^, [53]^2,3^, [54]^2,3^, [55]^2,3^	

^1^: Only in some cases

^2^: Additional background distinction by chemical analysis (filtration sample or single particle analysis)

^3^: Additional TEM or SEM analysis

^4^: Nearly clean room conditions: [4], not in table

Two approaches are represented by only one published study each (see Table 2). The approach based on comparative studies with and without nanomaterial was published only once [35] but according to the authors' personal communications is often used in industry. This approach assumes any handling and processing of the composite matrix without nanomaterial to be the point of comparison and any changes during handling or processing of the composite with nanomaterial can be attributed to the nanomaterial.

The "clean room approach" was not set up for the assessment of possible worker exposure but needed to avoid product material contamination by ambient dust. Nevertheless, the case in a facility producing carbon nanofibres which flushed a tent with nearly particle free air is an excellent example to avoid background particles from the outside. Still by-product particles either from the production process itself or from other sources such as electrical devices may influence the measurements.

Overall 25 studies related directly to workplace exposure were identified in the literature. More than 50% of these pursued the combined approach based on time series and spatial analysis. Quite often this approach was backed up by chemical and/or morphological analyses since none of the online devices for the detection of airborne nanomaterials is sufficiently selective to unambiguously determine product nanoobjects or their agglomerates. The combined use of the measurement devices significantly enhances possibilities for the distinction of nanoobjects from the background.

## 3. Exposure related workplace measurements

For this review, we have taken into account exposure related nanoparticle measurements at workplaces, where engineered nanoobjects and their agglomerates or products using nanomaterials are processed or used. These include industrial production facilities, processing plants, pilot plant investigations, crafting of nanomaterials (drilling, sawing etc.) as well as research related work area settings. Tables 3 and 4 summarize the reviewed articles. The work activities investigated can be structured along the production pathway: production, handling and refinement of the raw material, bagging and shipping of the nanomaterial, processing of the nanomaterial and work processes with the nanomaterial product. No strict separation according to types of nanomaterials (suspension or powders) is pursued but are indicated by the process investigated, e.g. production via the liquid phase.

**Table 3:** List of workplace processes and nanomaterials being investigated for possible exposure

Nanomaterial	Production	Handling & refinement	Bagging & shipping	Processing	
				
				Powder or suspension	In a fixed matrix
Carbon Black	[3]	[3,37]	[2,37]	[38]	

CNT, CNF, fullerenes	[4,33,46,48-51,54]	[38,33,4,46,45,50,49,54]	[4,45]	[38,45,50-52]	[42,52]

Ag	[41,55]	[41]		[33,53]	

TiO_2_	[39,56,55]	[39,33,56]			

SiO_2_	[39,43]	[39,43]	[36]	[43]	

Al_2_O_3_	[39]	[39]		[44,53,54]	[44,54]

Metals	[39,33,51]	[39,33]		[33]	[33]

Metal oxides	[39,43,33,45]	[39,43,33,45]	[45]	[43,33,45,47]	

Others	[39]	[39]		[40]	[40]

**Table 4:** Summary of the workplace related exposure studies

	Workplace	Type of activity	Nanomaterial	Metric	Results - remarks
[2]	Industrial production	Bagging areas of three plants	Carbon black	PSD(15-675 nm); MC; NC; CC	No significant release of nanoparticles detected, release of agglomerates (> 400 nm) of nanoparticles in all cases of bagging detected if open systems were used; Other sources also significantly influence nanoscale particle concentrations

[3]	Industrial production	Production and pelletizer areas of three plants	Carbon black	PSD(15-675 nm);MC;NC; CC	Significant release of nanoparticles (> 10^6 ^#/cm³) and their agglomerates detected in case of a leak in the pelletizing area; in case of good maintenance no significant release of NP from closed production and pelletizing processes; other sources significantly influenced particle number concentrations

[36]	Toner and printing inks industry	Bag emptying of powders	Fumed silica	NC (< 1 μm); PSD (< 1 μm); ASA (< 1 μm); morph.; CC	Significantly increased^1 ^NC (> 100 nm) and ASA detected during bag emptying; confirmed by TEM analysis

[37]	Industrial manufacturing plant	Manual packaging, warehouse, pelletizing	carbon black	PSD (< 1 μm); LDSA (< 1 μm)	Higher NC and LDSA concentrations during activity than during non-activity

[41]	Industrial manufacturing facility	Liquid phase process, drying, grinding, handling,	Silver	PSD (15 nm-675 nm); morph	Significant release of particles < 100 nm as well as of agglomerates was observed during all processing steps as soon as the reactor, dryer and grinder were opened, leading to possible exposure even for wet production processes

[45]	Industrial production	Metalloxide production (gas burner) and embedding into a porous oxide matrix, bagging, handling, cleaning and maintenance	MeO (no further information)	NC (10-1000 nm) PSD (14-760 nm), MC PM1 (0.1-1000 nm).	Long term study on possible release of nanomaterial; Significant release of nanomaterial by 'open' production line, handling and cleaning < 1000 nm; Increased NC < 100 nm concurrent with production activity.

[46]	Small commercial nanotechnology production facility	Production of fullerenes (arc reaction), sweeping, vacuum cleaning	Fullerenes	PSD (14 nm - 673 nm), PM2.5 MC, PAH MC	Slightly elevated NC in work area compared to background at one day out of 4 possibly related to cleaning of fume hood; Very good containment of the nanomaterial in the fume hood (production and handling area)

[47]	Industrial production	Wet mill	Lithium titanate metal oxides	NC (10-1000 nm), PSD (300 nm - 10 μm)MC (respirable fraction), CC, morph.	Only large agglomerates have been detected

[48]	Industrial production	Bagging and agitation including use of vacuum cleaner during these work steps	Fullerenes	PDS (15 nm-10 μm), morph	Release of particles < 100 nm were observed during bagging and vacuum cleaning; also release of particles > 2 μm was observed during all work steps, including agitation.

[50]	Industrial production	Production and processing (bagging, handling CNF in dryer, thermal treatment, removal from dryer)	Carbon nanofibers	NC; MC respirable; ASA; photoelectric response; CO and CO2	Elevated NC and MC indicate release of significant amounts of nanoscale particles and their agglomerates; no definite indication on release of single and agglomerated carbon nanofibres.

[51]	Industrial production pilot plant, Industry processing	Production and maintenance (silicon), extrusion of CNT nanocomposites	Silicon; CNT	PSD (5-600 nm); NC; ASA	No changes in PSD and NC was observed during production, but spikes during cleaning of mostly agglomerated silicon (> 200 nm); High NC concentration observed in the extrusion area, but no specific CNT detection method was employed;

[56]	Industrial manufacturing	Production, filtration, bagging	TiO_2_, Al_2_O_3_	PSD (5-600 nm); MC PM1; CC, morph	Wet and combustion production processes were compared and no significant release of particles < 100 nm observed; in one case a bag was overfilled and release of agglomerates > 400 nm observed

[55,61]	Simulated industry workplace	Compounding of nanocomposites with nanoscale alumina	Al_2_O_3_	PSD (5.6-560 nm), morph.	Significant release, confirmed by STEM analysis

[4]	Laboratory and industrial production facility	Normal activities during batchwise production of SWCNT: collection, removal, cleaning, opening container, vacuum cleaning	SWCNT	NC (10-1000 nm), MC (size fraction not indicated), morph, CC	Likeliness of CNT exposure during production given; period of exposure relatively short (ca. 1 h) but concentration are sometimes high the exposure nearly pure nanomaterial.

[33]	Laboratory to industrial workplace	Synthesis of nanoobjects, handling and production of composite materials	CNT, CNF, Carbon Nanopearls, fullerenes, TiO_2_, Ag, Mn, Co-oxide, Fe-oxide, Al, SiFe, QDs	NC (15-1000 nm) for screening, PSD (300-1000 nm), MC, CC (not size selective)	Increased NC in all three investigated size classes (10-1000 nm, 300-500 nm, 500-1000 nm) indicate Release of nanomaterial during various of the investigated sites; no systematic analysis of the results is presented

[38]	Research Laboratory for use of carbon based ENMs	Transfer of CNMs; sonication in environmentally relevant matrices	Fullerenes, MWCNT; carbon black	PSD (300 - 10,000 nm); NC (10 - 1,000 nm)	Each activity resulted in increased particle number concentrations; TEM images clearly show CNM

[39]	Research laboratories	Scalable flame spray pyrolysis	NaCl, BiPO_4_, CaSO_4_, Bi_2_O_3_, TiO_2_, SiO_2_, WO_3_, Cu/ZN, Cu/SiO_2_, Cu/ZrO_2_, Ta_2_O_5_/SiO_2_, Pt/Ba/Al_2_O_3_	PSD (15-675 nm); NC (> 7 nm; > 10 nm), MC (< 1 μm; < 10 μm)	Concentration in near field and far field higher than in background in 40% of measured cases

[40]	Research laboratories	Plasma enhanced CVD; PVD; compounding of polymers with nanofillers	Nanofillers (not further specified)	PSD (5 nm - 20 μm); NC (< 370 nm)	Increased concentrations detected, but likely not caused by ENP release

[42]	Laboratory scale production	Machining/cutting	CNT hybrid composites	NC, PSD (5 nm - 20 μm), morph, PM10 MC	Small increases in NC during wet cutting, significant increases (ca. 300,000 #/cm³) during dry cutting; fibres detected in concentrations of 1-4 fibres/cm³ during dry cutting.

[43]	Various^2^	Mixing of powder and liquid; filling/emptying oven; suspension spraying; flame spraying	TnO, ZnO, InZnO, SiO_2_	PSD (14 nm-20 μm), NC (< 1 μm), MC (respirable and inhalable)	No evidence of release of ZnO and InZnO during handling; very high concentrations during spraying of silane and flame spraying of SiO2 suspension

[49]	Laboratory scale production	Production by chemical vapour deposition (CVD)	SWCNT, MWCNT	PSD (5 nm - 20 μm); morph	SWCNT and MWCNT release was determined in the production area in the fume hood, depending on process conditions; No significant amounts of CNT were detected in the breathing zone of a worker and the background.

[52]	Laboratory scale production and handling	Weighing, mixing with solvent, cutting	raw CNF and CNF composite	NC (10-1000 nm); PSD (10 nm - 10 μm); ASA; morph	Slight increases in NC for weighing, mixing of CNF and wet cutting. TEM picture reveal the release of CNF during these processes. Minor airborne CNF concentration during normal handling; Main increase in PSD for sizes < 400 nm.

[53]	Laboratory handling	Handling in fume hoods of nanomaterial powders, pouring, transferring	Al_2_O_3_, Silver	PSD (5-600 nm); morph	increased NC in the breathing zone of a worker mainly in size range > 100 nm but also partially < 100 nm during handling activity;

[54]	Laboratory scale production and handling	Growth, removal, shaving and transfer of CVD derived CNT	CNT	PSD (5-600 nm); NC (10-1000 nm); TP, ESP; MC	Neither TEM nor NC analysis reveal a release of CNT during these processes

[62]	Laboratory and industrial production	Four production facilities (2 × TiO_2 _by combustion, Ag by plasma and in liquid via citrate), collection of powders in fume hood and in liquid	TiO_2_, Silver	PSD (15-710 nm); MC; CC, morph	Lowest number concentration detection for in liquid production, higher particle number concentrations during combustion but also release from electro engines and other side activities

### Production

Nanoobjects are either produced top-down by milling and grinding of bulk material or bottom-up starting from nucleation with subsequent particle growth by condensation and/or coagulation. The bottom-up process is the most common industrial synthesis route for nanoobject production. Two major process parameters significantly influence the possible release of nanomaterial: production via the gas [e.g. [51]] or liquid phase [41] and production in an open [45] or closed process [2,3]. Gas phase production process can further be differentiated into nucleation-condensation (metals), vapour deposition (CNTs) and gas-to-particle conversion via oxidation (metal oxides). The latter differentiation itself does not influence the possibility of release but may determine whether the process is open or closed (references given after the brief description of the process indicate corresponding exposure related studies).

### Handling and refinement

Once the nanomaterial is produced, it is removed from the process by filtration or opening of a reactor. Relevant, sometimes manual work steps are filtration [3], pelletizing [2], cleaning of raw nanomaterial [56], drying [41], grinding and milling [56].

### Bagging and shipping

The next working steps are bagging and shipment of the nanomaterial. The nanomaterial may either be handled as liquid suspension or dry powders. The latter is often discussed and investigated as a source for airborne exposure [2,4] while the prior significantly decreases the likeliness of airborne release. No investigations related to bagging/filling and shipping of suspensions have been published so far.

### Processing

The area of activities related to processing of nanomaterials covers a wide range such as mixing nanomaterial powders in liquids, cutting, drilling etc. of composites, drying and spraying. Therefore one single study can rarely cover all processing activities of one nanomaterial. E.g. the handling and mixing of CNF was investigated by Mazzuckelli et al. [52] but similar processes for TiO_2 _or other nanomaterials are not published. We may differentiate processing steps with the nanomaterial itself as a powder or in a suspension and those processes, e.g. drilling or cutting, when the nanomaterial is embedded in a matrix.

### Work process with nanomaterial products

The use of nanomaterial coated products at medical workplaces, workplaces using nanomaterial products, such as dry cleaners, parquet sanding are also nanomaterial related workplaces. Exposure scenarios towards the nanomaterial for these workplaces have so far not been published to our knowledge and are not further covered in this review.

An overview on which process was investigated including which materials is given in Table 3. It can be seen that most of the materials and processes have been investigated. Still quite a few processes were only part in overview studies, e.g. by Methner et al. [33] or Möhlmann et al. [43], which summarize results but do not go into detail and give no clear results whether the actual nanomaterial was released or not.

The lack of a harmonized approach concerning measurement strategies and techniques, metrics and size ranges as well as the data analysis procedures complicates the summary of the studies listed in Tables 3 and 4. The different measurement strategies and measurement devices were introduced in the prior section. From Table 4 it is evident that all studies used particle number concentrations (NC), either directly determined by a CPC or derived from particle size distribution (PSD) measurements, in their analysis for possible exposure. This is not surprising since number concentration measurements are relatively easy, cheap and very sensitive towards nanoparticles. The main problem for a combined assessment of health effect studies related to NC and PSD is that no defined lower detection limits for particle size are used. This leads to difficulties in the comparison of absolute values, especially in case of number concentrations, where contributions of sub-10 nm particles can be significant.

21 out of the 25 studies summarized here also determined the particle size distribution down to below 100 nm particle size. This is seen to be essential to actually allow for the differentiation of larger agglomerates from the more mobile particle size fraction below 100 nm. While the time resolution of NC measurements is normally relatively high (1 Hz), time resolution of PSD devices may become crucial when studying processes with quickly changing aerosols. It is also important to note that determined size distributions, either by electrical mobility or optical properties are based on diameters of electrically or optically equivalent spheres, which may not be very meaningful for non-spherical particles.

Slightly more than half of the studies determined also the mass concentration (MC). Various devices, e.g. optical particle sizer based measurements, stationary and personal filtration samplers, and PM1, PM2.5, PM10 samplers were employed. The use of filtration samplers and MC determination is seen to be important to enable the linking with conventional workplace exposure assessment and legislative limits. Nevertheless, this metric is dependent on the particle volume, i.e. particle diameter to the third power and is hence very insensitive to nanomaterials in the lower submicrometer size range.

The importance of the use of electron microscopy (EM) for nanomaterial exposure related studies is stressed by the fact that 12 studies included this time consuming analysis. Even though the use of EM may not be seen as quantitative it is still the only method, which allows for a definitive identification of product nanomaterial, especially when linked with single particle chemical analysis. Good examples are here the detection of CNTs and CNFs.

Eight studies also used consecutive chemical analysis to derive more information on whether the airborne material may have been the nanomaterial in use. Bulk chemical analysis can be used for the different size fractions as indicators, but only single particle analysis can provide definitive differentiation from the background.

The possible importance of the particle surface as exposure metric was presented by Oberdörster et al. [57] and stressed by several other researchers [58,59]. Easy to use, portable devices became available only in recent years an4 studies already used these devices. None of the currently available measurement devices measures the geometric surface area of particles, but the lung deposited surface area or "active" surface area. Although the delivered metric is not the geometric surface area of the particles, the devices output can directly be linked to health relevant toxicological reaction mechanisms. The practicability is a further advantage of this measurement technique. On the other hand, this metric is increasingly insensitive for decreasing particle sizes.

The lack of any uncertainty and detection limit discussion in all of the studies is another problem in the assessment of the published exposure related studies at workplaces. Kuhlbusch et al. [56] have recently published a first uncertainty estimate for the method they employed. They showed that no single uncertainty value can be given due to the high dependency on local conditions, like variance in background concentrations. If two or more devices are used simultaneously to obtain a spatial distribution, the uncertainty is furthermore influenced by the measurement accuracy and intercomparability of the measurement devices. Even two devices of the same type can show deviations up to approximately 30% [60,44].

Some general conclusions on the focal point, possible airborne exposure to nanomaterials, can be drawn from the studies. 22 of the 25 studies indicate release of nanomaterial particles > 100 nm. The remaining three studies reported increased values in the work area but stated that the elevated concentration may not be attributed to the nanomaterial. Two of those were laboratory scale production studies related to CNTs. This ambiguity in the identification is much more common for the reported values for particles below 100 nm particle size. 13 of the 25 summarized workplace studies in Table 4 indicate release of nanomaterial below 100 nm particle size. But, only few studies were able to clearly identify the nanomaterial, Kuhlbusch et al. [3], Fujitani et al. [48], Tsai et al. [49]. In other cases, results were much less clear as in Yeganeh et al. [46] and Evans et al. [50]. This listing shows that the identification of nanomaterial exposure is not straight forward and that well described and harmonized methodologies are needed a) to unambiguously link elevated particle concentrations to the nanomaterial under investigation and b) come to quantitative results. The latter is extremely difficult and possibly needs process studies on the laboratory scale.

## 4. Nanoparticle release studies under laboratory conditions

Systematic and quantitative analysis of processes and materials can be performed under defined boundary conditions in the laboratory. Therefore, potential sources must be previously identified and selected. Relevant process-related parameters have to be chosen for the transferability to real exposure situations. To meet these requirements, some studies used standardized procedures for the experimental process simulation [53,63-66]. Due to the multitude of processes which lead to the release of particles, the number of studies on new characterization methods is steadily increasing [29,54,66,67].

Process control (e.g. avoidance of background concentration and particle contamination, sampling), conditioning of the generated aerosol (e.g. dilution, neutralization, size selective particle deposition), and the combination with suitable measurement technology are crucial for the quality of investigations on particle release in the laboratory. Particle number based aerosol measurement devices, originating from clean room monitoring, enable the highest sensitivity for the quantification of low particle amounts. Therefore the nanoparticle release is expressed as a particle number released from a sample of defined size [68].

Nearly all of the reviewed laboratory studies are based on reducing background concentrations. The easiest way to ensure a stable and well known background concentration is to isolate the test room atmosphere from outside influences as performed by Bello et al. [42,54,69]. Lower background concentrations require encapsulation and purging with filtered air or gas as realized by the most studies. The lowest background concentrations were achieved by using laminar flow benches which also provide larger working space as employed by Vorbau et al. [64] and Göhler et al. [67].

To ensure the comparability of different tests or to estimate the relevance for a certain workplace situation the volumetric flow over the treated sample, the analysed volumetric flow of the measurement device and the dilution ratio must be measured to calculate the number of emitted nanoparticles (defined as nanoparticle release) from the measured particle number concentration and particle size distribution. Furthermore, the comparability requires the reporting of treated sample size, for instance sample mass. Relating the released nanoparticle number to this sample mass delivers a kind of emission factor of a certain material in a certain treatment process.

Generally, nanomaterial release studies can be structured in following steps:

▪ reducing background particle concentration

▪ sample treatment and/or process simulation

▪ nanomaterial release and aerosolisation

▪ aerosol sampling and conditioning

▪ aerosol/particle characterization

Nanomaterials are subjected to mechanical, thermal and environmental stress situations during production, processing and use. Published studies, based on the characterization of the particle release into air due to individual treatment processes, can be roughly classified by the investigated nanomaterial group (powders, suspensions, coatings, composites) as shown in Table 5. According to these groups the experimentally simulated treatment processes (shown in column two) are different like fluidized bed processes for powders or sanding processes for coatings. Comparable processes are separated additionally. Moreover, the processes are ordered downwards with increasing energy input.

**Table 5:** Studies focused on the particle release of ENMs by laboratory testing (column 1 indicates the different subcategorisations)

Study	Process	Materials	Instrumentation	Metric	Results
*Powders*					
[53]	Rotating drum test (free fall, stirring, ...)	TiO_2 _(Aeroxide P25), ZnO	SMPS, APS, MOUDI	PSD (15 nm - 20 μm), NC, DI	< 10% particles < 100 nm60% particles < 1 μm
[65]	Rotating drum test (free fall, stirring, ...)	TiO_2_, SiO_2_, FeO(OH), Mg_3_Si_4_O_10_(OH)_2_, Al_2_O_3_	FMPS, APS, Filtration	PSD (5.6 nm - 20 μm), NRP (0.5 μm - 20 μm), NRP (5.6 nm - 560 nm), DI	Undefined fraction of particles < 100 nm
[66]	Rotating drum test (free fall, stirring, ...)	Organoclay, Bentonite	FMPS, APS, Filtration	PSD (5.6 nm-20 μm), NRP (0.5 μm - 20 μm), NRP (5.6 nm - 560 nm), DI	Undefined fraction of particles < 100 nm
[70]	Free fall	TiO_2 _(G5), SiO_2 _(Aerosil 200)	ELPI; SEM (ELPI)	PSD(30 nm - 10 μm), MC, MC/M	Fraction of particles < 100 nm

[71]	Fluidized bed	TiO_2 _(Aeroxide P25)	SMPS (LDMA, NDMA), APS; TEM (ESP)	PSD (4 nm - 20 μm)/NC_max_	10% particles < 100 nm 70% particles < 1 μm
[4,72]	Vortex shaker (fluidized bed, agitation)	SWCNT, alumina powder	SMPS (LDMA, NDMA), APS	PSD (4 nm - 20 μm), NC	Fraction of particles < 100 nm, alumina powder released more NP than SWCNT
[73]	Vortex shaker (fluidized bed)	SWCNT, MWCNT, TiO_2_, ZnO	SMPS, HHCPC, APS, OPC	PSD (10 nm - 20 μm), NC (10 nm - > 1 μm)/V, NC (10 nm - > 1 μm)/M	Fraction of particles < 100 nm
[74]	Shaker method	MWCNT	SMPS, APS, TEM(ESP, CI)	PSD (14 nm - 20 μm), NC(dt)	Fraction of particles < 100 nm; peak at 200-300 nm
[75]	Fluidized bed with oscillating sieve plate	MWCNT	SMPS, SEM (TP)	PSD (< 1 μm), NC(dt)	Fraction of particles < 100 nm

[29]	Stirring and dispersing in orifice (leak in pressurized vessel)	TiO_2_, CeO_2_, SrCO_3_, TiZrAlO	SMPS	PSD (14 nm - 736 nm), fractions, relative values	Increase of the fraction of ENPs by increase of the overpressure (up to 12%)

*Suspensions*					
[38]	Weighing/transferring of powders and sonication of suspensions	fullerenes, MWCNT, CB	HHCPC, HHPC, TEM-EDX (filtration)	PSD (0,3 μm - 10 μm), NC (10 nm - 1 μm)	Suspension sonication leads to droplets with embedded ENM
[77]	Spraying	suspensions with and without Ag	SMPS, TEM(ESP)	PSD (10 nm - 500 nm), NC (< 100 nm), NC(< 500 nm)	High fraction of particles < 100 nm

*Coatings*					
[63]	Weak abrasion process (Taber Abraser)	PVC layer with/without ENPs (nanoclay)	SMPS, CPC	PSD (5 nm-1 μm)	
[64]	Weak abrasion process (Taber Abraser)	coatings with/without ENPs (ZnO)	SMPS, CPC; SEM/TEM-EDX (ESP)	PSD (16 nm - 626 nm), NC (> 6 nm), wear mass	Very low concentrations, ENPs still embedded

[81]	UV light, wind erosion, scrabing	coatings with TiO_2_	SMPS	PSD (15 nm - 661 nm), NC (15 nm - 661 nm)	Comparison with non-doped samples is missing
[54]	Shaving (razor blade)	CNT	FMPS, HHCPC, SEM & TEM - EDX (TP)	NC (10 nm -1 μm), PSD (5.6 nm-560 nm)	No significant change in concentration; no free CNTs were observed

[7,8]	Sanding process (orbital sander)	coatings with/without ENPs (TiO_2_, CB, SiO_2_, CaCO_3_)	FMPS, APS(ESP)	PSD (5.6 nm-20 μm)	General release of NP, spark particles contamination
[67]	Sanding process (Dremel)	coatings with/without ENPs (ZnO, Fe_2_O_3_)	FMPS, CPC, OPCSEM/TEM-EDX (ESP)	PSD (5.6 nm-20 μm), NC (< 100 nm), NRP (< 100 nm), NRP (< 10 μm), swarf mass; material, morphology	General release of NP but ENPs still embedded in the matrix

*Composites*					
[82]	UV-light; weak abrasion process (Taber Abraser); customized sanding	POM with/without CNT, PA with/without SiO_2_, cement with/without CNT, cement with/without CSH	SMPS, UNPA, SEM, AUC, XPS, SIMS	PSD (14 nm-820 nm), Morphologie, material	No free SiO_2_-particles or CNTs detected
[42]	Dry and wet cutting (band-saw; rotatory cutting wheel)	composites with and without CNT	FMPS, APS, HHCPC, DT, SEM/TEM-EDX (ESP, TP)	PSD (5.6 nm-20 μm), NC (5.6 nm - 560 nm), NC (0.5 μm - 20 μm) MC, material, morphology	No free CNTs observed
[69]	Dry and wet solid core drilling	Composites with and without CNT	FMPS, APS, HHCPC, DT, DC, SEM/TEM-EDX (ESP, TP) WRASS+ICP-MS	PSD (5.6 nm-20 μm), NC, MC (< 35 μm), SA; material; morphology	Smoke generation, free CNT clusters observed

### Powders

Based on the various application processes (e.g. transport, dosing, filling) of powders and their characteristic parameters (e.g. energy input, flow velocity, sample amount), different methods have been developed in the past for the investigation of material dependent particle release, the so called dustiness. Additional material dependent parameters, like powder flow properties also affect the results of such dustiness tests and limit their comparability. Nevertheless different types of test setups are described in national standards and two test procedures based on different drop conditions (rotating drum test, continuous drop test) were introduced as international standard (EN 15051:2006). These methods, developed for MC assessments, were taken, modified and combined with aerosol measurement devices by Schneider & Jensen [65], Jensen et al. [66] and Tsai et al. [53] for the characterization of the particle release. Schneider & Jensen [65] and Jensen et al. [66] investigated the particle size distribution and particle number concentration of different metal oxides powders (TiO_2_, SiO_2_, FeO(OH), Mg_3_Si_4_O_10_(OH)_2_, Al_2_O_3_), bentonite and organoclay powders. Typical results show a number based submicrometer fraction of about 60% and < 10% for particles < 100 nm. Similar results were obtained by Tsai et al. [53] for TiO_2 _and ZnO metal oxide powders with slightly modified measurement devices (SMPS, APS, and MOUDI). Isbaseta & Biscans [70] investigated TiO_2 _and SiO_2 _in free drop conditions. Despite large deviations in the PSD determined by an ELPI, both powders show a release of nanoparticles of 10 mg·m^-3 ^an40 mg·m^-3 ^for TiO_2_.and SiO_2_, respectively, for the same initial sample mass.

Studies based on fluidized bed processes represent a second test type for dustiness. Maynard [71] aerosolized TiO_2 _powder in a two component fluidized bed to determine the agglomerate size distribution between 5 nm and 20 μm. Furthermore, airborne particles were electrostatically-precipitated for subsequent TEM analysis. Results showed about 10% of the particle number concentration to be < 100 nm diameter and 70% in the submicrometer range. Baron et al. [72] and Maynard et al. [4] introduced a new method for agitating CNTs and metal oxide powders to achieve higher particle concentrations. Powder samples or mixtures of powders and beads were filled in a HEPA-filtered air purged shaker-agitated centrifuge tube. The PSD measurements (3 nm - 20 μm) showed that fumed alumina powder released more particles < 100 nm than the investigated SWCNT powder. The agitating principle of Baron et al. [72] and Maynard et al. [4] was also used by Ogura et al. [73] to characterize the amount, PSD and morphology from different nanostructured powders (TiO_2_, ZnO) and carbon materials (SWCNT, MWCNT, fullerenes). In addition to the PSD, Ogura et al. [73] relate the measured number concentrations to the applied sample volume or sample mass. Lee et al. [74] used a similar shaker-principle for the PSD characterization of MWCNTs. They also employed TEM-analyses. Results of atomized, dried and neutralized suspensions of the same MWCNT and ultra-pure water show similar peak diameters at approximately 200 nm up to 300 nm in comparison to the shaker-generated aerosol. In each case fractions of particles in the nanometer size range were observed. An extension of the fluidized bed to aerosolize CNTs was presented by Plitzko et al. [75]. To improve the aerosolisation process, the sieve plate of the fluidized bed was agitated by a shaker. First results based on SMPS measurements and TEM investigations on TP-precipitated particles show a considerable release of CNTs with material specific fractions of particles/agglomerates in the nanometer size range. The amount of particles with electrical mobility diameters < 100 nm was determined to be between 10% and 60% of the determined number based particle size distribution < 1000 nm.

The procedures and results presented up to here are based on a low energy input to the powder. High energy processes, on purpose or by accident, can result in significantly higher energy input to the nanomaterial. Therefore, worst-case scenarios have to be investigated. The particle release due to a leak in a pressurized vessel was experimentally simulated by Stahlmecke et al. [29]. Different metal-oxide powders (TiO_2_, CeO_2_, SrCO_3_, TiZrAlO) were aerosolized using a pressurized beaker in combination with a magnetic stirrer and passed through an orifice. Results are given as PSD and relative concentrations based on the comparison with reference measurements of powders dispersed without orifice, mimicking the leak. In the measured size range < 736 nm, the amount of particles < 100 nm of the number weighted particle size distribution increases from 1% up to max. 12% with increasing overpressure from 0 kPa to 140 kPa, depending on the material. Another worst case scenario, preferred by NIOSH and introduced in ISO DIS 12025:2010 [68], is the method described by Boundy et al. [76], where a small amount of powder - aroun4 g - is completely sucked and dispersed in an evacuated bottle.

Nearly all powder studies suffer from incomplete determination of the energy input during sample treatment. Some powder drop tests were affected by the material properties, e.g. flowability, themselves. Furthermore repeated treatment of powders in rotating drum or fluidized bed can generate secondary agglomerates. In this case the number of released particles rapidly decreases during the test [72,75]. Fast measuring devices may help to avoid this problem [65,67]. Furthermore, treatment processes stressing a well-defined sample only once allow a straight correlation of the measurement data to the treatment process. Repeated energy input leads to permanent changes in the experimental conditions.

### Suspensions

Johnson et al. [38] investigated the particle release of fullerenes, MWCNT and carbon black into air due to the sonication of suspensions in comparison to different laboratory handling processes, especially the weighing and transferring of powder material. Size specific particle number concentrations (300 nm - 10 μm) and total particle number concentration (10 nm - 1 μm) were analysed. In addition, the generated aerosols were filtrated for subsequent TEM analysis. Johnson et al. (2010) [38] found that sonication leads to the release of water droplets with embedded nanomaterials. CPC measurements showed an increase in concentration by sonication in comparison to the dry powder bulk handling, while OPC measurements showed a decrease in the concentration of coarse particles. It remains unclear whether all droplets contained nanomaterial.

Spray simulation with nano-silver and nanoparticle-free suspensions were carried out by Hagendorfer et al. [77] inside a particle free glove box. Two different types of commercial spray dispensers (pump spray, propellant gas spray) were investigated. The generated aerosols were dried by a thermodesorber before PSD measurement and sampling by an ESP for subsequent TEM and EDX analysis (0.8 m away from the generation zone). The pump spray experiments showed no significant increase in the number concentration (10 nm to 500 nm) in comparison to the background. This can possibly be explained by the formation of only large droplets which deposited inside the glove box before sampling. Propellant gas spray, on the other hand showed a broad PSD. The silver particle sizes, determined with SMPS and TEM, depended on the droplet size generated by the dispenser. The measured number based PSDs of the propellant gas spray generated aerosol show a fraction of around 80% < 100 nm.

### Coatings

Engineered nanoparticles are often intentionally embedded in the matrix material of a coating, which can be understood as a thin layered composite on a substrate. Several studies were performed to determine potential exposure of consumers by typical application processes of such coatings like abrasion, scraping or sanding. Besides the determination of the particle concentration and the PSD, additional appropriate chemical and morphological analyses (e.g. SEM, TEM, EDX, ICP-MS) are necessary for the distinction of unintentionally produced particles in the nanoscale and the engineered nanoobjects in the airborne emissions.

Vorbau et al. [63] employed a commonly used and standardized Taber Abraser (DIN 68861-2:1981 [78], DIN EN 13523-16:2005 [79], DIN EN ISO 7784:2006 [80]) for determining the particle release from different coatings with and without ZnO nanoparticle additives. This device consists of two abrasion wheels, which act on the sample surface. Investigations were performed by operating the Taber Abraser in a particle free environment inside a laminar flow bench. Due to the weak abrasion process, the nanoparticle concentration was too low for determining PSDs by SMPS. The average total particle concentration based on the CPC measurements ranged between 1 cm^-3 ^to 20 cm^-3^. No significant differences were observed between coatings with and without added nanomaterial. TEM images of electrostatically collected wear particles showed the nanomaterial only embedded in the matrix. In another study Guiot et al. [63] used the Taber Abraser test method for investigations on polyvinylchloride layers with and without nanoclays on a PET substrate. In contrast to Vorbau et al. [64] the generated and aerosolized particles were locally sampled near the abrasion zone. The abrasion zone was encapsulated and purged by HEPA-filtered air. Measurement data show an increase in the particle concentration for nanoclay-doped samples. The authors subtracted the measured PSD of the PVC layer without nanoclays from that with nanoclays. This calculation assumes same particle release rates in both experiments for balancing particle amounts, which was not shown. Furthermore, the difference in the released number of nanoparticles may not represent the nanoclay release due to changed mechanical matrix properties introduced by the addition of nanoclay.

Nanoparticle release due to light at different wavelengths, air flow and mechanical scraping for surface coatings containing nanomaterials were investigated by Hsu and Chein [81]. Coatings with different TiO_2 _additives on wood, polymer and tile were tested in a closed, purged chamber and PSDs were measured (15 nm - 616 nm). The highest particle concentrations of about 630 cm^-3 ^were found for the coating on tile and wood during parallel use of UV-light, airflow and scraping. No comparative measurements of coatings without added nanomaterial were performed, so a clear interpretation of the measured data is not possible. Bello et al. [54] investigated the particle release during the removal of CNT forests, which were artificially grown by CVD on a silicon substrate, by cutting with a razor blade. No significant difference between background concentration and material handling was observed using an FMPS and a CPC. SEM and TEM-images of electrostatically and thermophoretically collected and filtrated aerosol particles showed typical background particles but no CNTs, whether individual or bundles.

A commercial hand-held orbital sander with an internal dust removal fan was operated by Koponen et al. [7,8] for the characterization of the sanding dust. The exhaust air of the sander was connected to an exposure chamber for determining the PSD (5 nm - 20 μm). Additionally, particles were electrostatically sampled for subsequent physicochemical and toxicological analysis. Coatings containing and not containing engineered nanoparticles were investigated. Some coatings showed higher others lower release rates when engineered nanoparticles were added to the coating. Sanding of surface coatings with and without ZnO and Fe_2_O_3 _nanoparticle additives was investigated by Göhler et al. [67]. The sanding zone was encapsulated and a particle free environment was provided by a laminar flow bench. PSD (5 nm - 20 μm) and number concentrations (CPC) were measured and ESP samples analysed by SEM, TEM and EDX. Despite a considerable generation of nanoscale particles due to the sanding process, neither matrix-free engineered nanoparticles nor significant differences in the particle size distribution between coatings with and without engineered nanoobjects were observed in both studies.

### Composites

Particle release due to Taber Abraser tests, customized sanding and UV-light weathering was compared by Wohlleben et al. [82] for different composites containing SiO_2_, CNT, CSH nanomaterial. In each case the morphology, the size distribution and surface chemistry of the wear powders were analysed. Aerosol particles were measured by SMPS for the first two processes. Only the polymer-composite with CNTs behaved significantly differently to the same material without nanoobjects, where increased UV-light adsorption resulted in faster matrix degradation.

The ability for the release of CNTs from composite materials was also investigated by Bello et al. [42,69] during wet and dry cutting and solid core drilling. In both studies the same types of CNT-composites were analysed. Bello et al. [42] used a band saw for the simulation of a dry cutting process, while a rotating cutting wheel was operated for the wet cutting process. Samples for the analyses were taken at two different locations - near the source for the dry cutting process and at the breathing zone. The PSD (5 nm - 20 μm), NC (10-1000 nm) and MC (PM2.5, optically determined) were measured. Analysis for particle chemistry and morphology were carried out by SEM, TEM coupled with EDX on ESP and TP substrates. A considerable generation of nanoscaled particles during the dry cutting process was observed. Results show no significant difference between samples with and without CNTs with regard to the particle number concentration. SEM and TEM analysis showed no free CNTs and no CNTs which are bundled or attached to coarser particles.

A commercial drill press was employed by Bello et al. [69] to simulate dry and wet solid core drilling. The latter one was simulated by continuous spraying of deionized water to the drilling zone. In comparison to Bello et al. [42] a WRASS was added to the measurement devices in [69] for size selective particle deposition and subsequent ICP-MS analysis. During the drilling processes smoke generation was observed. Also clusters of CNT were determined with TEM. Whether these were released directly from the drilling process or originate from contaminations remains unclear.

## 5. Discussion and Conclusions

Two different basic approaches to derive information on possible workplace exposure were summarised and the state of the art presented in this review. It is evident that both approaches, workplace measurements and laboratory studies, are needed for a concise assessment to derive e.g. model based predictions on possible release of nanomaterial from a given process.

All studies can be ordered in a systematic scheme of different workplaces, work processes and nanomaterials. This systematic evaluation shows that all basic parameters have been pursued and published in the peer-reviewed literature. Thus, the amount of data becoming now available will allow first meta-analysis investigating material structure and release relationships in detail. Despite the major progress made in recent years, severe open issues still exist hindering the set-up of a coherent and concise exposure assessment for nanomaterials at workplaces. A coherent approach for the likeliness of release and workplace exposure assessment for all relevant nanomaterials and work processes as e.g. outlined in Table 3 is recommended.

Measurement metrics and corresponding measurement technology remain to be an extremely important issue. The metric best related to possible health effect is still not identified. The lack of comparability always became evident, when trying to compare results from the various studies in this review, because a harmonized approach for data evaluation concerning metric, size range etc. is still missing. This lack hinders the development of general conclusions.

Particle number concentrations and particle number size distributions are the most commonly used metrics within the reviewed workplace and laboratory studies. This approach is currently seen as the one to further develop due to the high sensitivity of the metric to airborne nanoobjects as well as due to the availability of the measurement devices. The latter allow the use in tiered approaches starting with a screening e.g. only measuring a size-integrated concentration quantity such as the number concentration. Intensified measurements can be conducted in a second tier in identified areas of increased concentration.

A major drawback of current state of the art measurement devices is their lack of differentiation of background from nanomaterial related particles. Aerosol mass spectrometer is currently the only instrument sizing and chemically analysing nanoscale particles online. Such instrumentation, once it is capable of also analysing metals and metal oxides, which is currently in development, would be an ideal analytical instrument to be used for areal measurements at workplaces if costs and size of the instrument are neglected. On the other hand particle number concentration and particle size distribution measurements at workplaces can at best only be viewed as indicative measurements for the presence or absence of airborne nanomaterials but without a definitive proof. The latter can only be provided by particle sampling with subsequent electron microscopy, if possible linked with a single particle chemical analysis.

A second drawback may be the sensitivity and comparability. While CPCs can reliably measure concentrations down to 0.001 cm^-3^, size resolved measurements for nanoscaled particles need significantly higher concentrations. In addition, the commonly noisy background makes it impossible to detect a release which only amounts to a small fraction of the total concentration. In case of the need for detection of concentration increases of a few particles per cm³ other devices and methods have to be employed. An obstacle related to this topic is the current lack of information of lower and upper detection limits related to measurement devices and especially when combined with a measurement strategy. Further uncertainty arises from the calibration of the devices which sometimes may differ by up to 30% [60]. Work on the uncertainties and detection limits is certainly urgently needed to achieve data qualities good enough for comparison of results from different studies.

The method mostly used for the identification of nanomaterials is electron microscopy (SEM or TEM) coupled with single particle chemical analysis such as EDX. This method was regularly employed in different studies but is not standard in routine workplace assessments due to the high demand of person hours. A difficulty for this approach is the limitation of the measurements. Nevertheless the use of single particle analysis, combining morphological and chemical information, is currently the only approach proofing the presence of engineered nanomaterials. A definitive proof of their absence may not be possible due to the limitation of the subsequent quantification to a few thousand particles, even if automated particle identification software is used.

There is still an urgent need for a systematic approach of harmonization and standardization. The needed areas to be covered are test procedures simulating workplace activities and processes as well as coherent workplace exposure assessments. First steps in simulating workplace activities and work processes are investigations on sensitivities of nanomaterial emissions to specific work parameters such as in sanding on the type of sanding paper, the acting force onto the surface, rotational speed. Further steps in exposure assessments is the European, better worldwide, agreement on a harmonized measurement metric, strategy, and data treatment and analysis including statistics.

From the discussion above and the outcomes of the studies presented in this review, we conclude that a tiered approach is viewed as most practical for workplace and laboratory measurements, because complete measurement campaigns are very time and hence cost intensive and may only be necessary if there is evidence of an increased particle concentration. The proposal brought forward by NIOSH [33] and the consent report [34] are scientific reasonable and pragmatic starting points for further refinements. Laboratory studies are the consequence of the tiered approach.

Some studies reported a release of particles. In fewer studies engineered nanoscale particles were observed. The latter was mainly caused by maintenance problems, open gas phase production processes, open handling of nanopowders or smoke generation during processing.

A release of agglomerated nanoobjects, mainly > 300 nm in the number weighted diameter, was regularly observed, especially during open handling of dry nanomaterials [2,3]. Release of nanomaterials < 100 nm was only observed in the few special cases mentioned above. The use of fume hoods and appropriate ventilation systems seemed to significantly reduce potential exposure concentrations.

The review of the laboratory test procedures shows that many workplace related processes are currently simulated in the laboratory. While some approaches are quite advanced we still lack a coherent, systematic approach over all work related processes as well as studies on single simulations. Questions like: How shall abrasion tests be pursued? How does the normal force of the abraser influence the release? Shall heating of the sample be kept at a minimum? Shall the test simulate worst case scenarios? How to avoid background particles? have to be answered before a simulation method can be performed. This area, important for modelling of work processes and subsequent possible exposure, has to be further developed on the basic research level as well as in view of a standardized method.

Generally all reviewed studies underline that the amount of released nanoparticle is the result of the combination of the treatment process and the employed material. All powder handling processes released some nanoobjects. Release of free engineered nanoparticles was not observed in laboratory studies of workplace related processes based for treatment processes on coatings and composites because the nanomaterial was still embedded in the matrix material.

Still one of the major issues to be tackled in the near future is the question of how to detect and define level of detection needed for workplace safety assessment. In the case of a specific health hazard of a nanomaterial, lower detection limits may be down to single particles are necessary. Generally, particle number concentration based exposure values may be sufficient in most cases. When assessing the metric to be used for limit values, possible other parameters, such as particle surface area and particle reactivity [e.g. [83]], have to be evaluated. The use of mass concentration measurements is currently viewed as being too insensitive to assess toxicological effects related to airborne nanomaterials.

A final point revealed by this review is that a certain set of minimum information is needed for all workplace related studies, either real workplaces or laboratory simulations. Release assessments and comparability between different studies require sample size specific nanoparticle release data, which can only be obtained by simultaneous measurement of several more parameters than simply particle size distributions and number concentrations. Nevertheless, in each case, whether workplace studies or laboratory investigations, comparisons with reference activities, reference materials or different treatment processes are of fundamental importance for the discussion on possible nanomaterial exposure. The nanomaterials and processes studied have to be described carefully and in sufficient detail, including contextual information, to be able to compare the results with those of other studies. All methods employed to identify the corresponding nanomaterial should be described and a clear conclusion given if a release or exposure was determined. A last but very important piece of information is the reporting of particle sizes and size resolved concentrations. Integrated particle number and surface area concentrations alone may be used as indicators in a tiered approach, but do not describe exposure in sufficient detail.

## List of abbreviations

APS: Aerodynamic Particle Sizer; AUC: analytical ultracentrifugation; CNF: carbon nanofibers; CNT: carbon nanotubes; CPC: Condensation Particle Counter; CSH: calcium silicate hydrate; DI: Dustiness Index; DT: Dust Track; EDX: energy dispersive X-ray spectroscopy; EEPS: Engine Exhaust Particle Sizer; ELPI: electrical low-pressure impactor; EM: electron microscopy; ENM: engineered nanomaterial; ENP: engineered nanoparticle; ESP: electrostatic precipitator; FMPS: Fast Mobility Particle Sizer; HHCPC: hand-held condensation particle counter; HHPC: hand-held particle counter; ICBA: International Carbon Black Association; ICP-MS: Inductively Coupled Plasma - Mass Spectrometry; M: mass; MC: mass concentration; MOUDI: micro-orifice uniform deposit impactor; NC: number concentration; NRP: number of released particles; NSAM: Nanoparticle Surface Area Monitor; OPC: Optical Particle Counter; PM: particulate matter; PSD: particle size distribution; SA: Surface Area; SEM: scanning electron microscope; SIMS: secondary-ion mass spectroscopy; SMPS: Scanning Mobility Particle Sizer; TEM: transmission electron microscope; TP: thermophoretic precipitator; UNPA: universal nanoparticle analyzer; V: volume; WRASS: Wide Range Aerosol Sampling System; XPS: X-ray photoelectron spectroscopy

## Competing interests

The authors declare that they have no competing interest, neither in financial nor in non-financial regard. The researchers from IUTA and from TUD collaborate with industries in several publicly (EU, BMBF, BMU, BMWI) and non-publicly funded projects.

## Authors' contributions

All authors were involved in the initial discussion on the general outline of the manuscript based on the initial outline from TK, MS and DG. Literature collection, distribution and establishing the first overview was done by all authors. The key structure for the evaluation of the sections measurement strategy and workplace exposure measurements was set by CA and TK, for release studies under laboratory conditions by DG and MS. TK wrote the draft of the sections 1, 2, 3 and 5, while DG and MS drafted section 4. All sections were commented, improved and discussed between all authors. All authors read and approved the final manuscript.

## Endnotes

^1 ^The wording and definitions used in this review article is based on ISO/TS 27687 (2008) [84].

## References

[B1] Oberdörster G Ferin J Finkelstein J Wade P Corson N "Increased pulmonary toxicity of ultrafine particles? II. Lung lavage studies." J Aerosol Science 1990 21 384 387 10.1016/0021-8502(90)90065-6

[B2] Kuhlbusch TAJ Neumann S Fissan H Number size distribution, mass concentration and particle composition of PM1, PM2.5, PM10 in bag filling areas of carbon black production J Occup and Environ Hyg 2004 1 660 671 10.1080/15459620490502242 15631057

[B3] Kuhlbusch TAJ Fissan H Particle characteristics in the reactor and pelletizing area of carbon black production J Occup and Environ Hyg 2006 3 558 567 10.1080/15459620600912280 16998988

[B4] Maynard AD Baron PA Foley M Shvedova AA Kisin ER Castranova V Exposure to carbon nanotube material: Aerosol release during the handling of unrefined single walled carbon nanotube material J Toxic And Environ Health 2004 67 87 107 Part A 10.1080/15287390490253688 14668113

[B5] *ISO/TR 27628* Workplace atmospheres - Ultrafine, nanoparticle and nano-structured aerosols - Inhalation exposure characterization and assessment 2007

[B6] Szymczak W Menzel N Keck L Emission of ultrafine copper particles by universal motors controlled by phase angle modulation J Aerosol Sci 2007 38 5 520 531 10.1016/j.jaerosci.2007.03.002

[B7] Koponen IK Jensen KA Schneider T Sanding dust from nanoparticle-containing paints: physical characterisation J Phys: Conf Ser 2009 151 012048

[B8] Koponen IK Jensen KA Schneider T Comparison of dust released from sanding conventional and nanoparticle-doped wall and wood coatings J Expo Sci Env Epid 2010 1 10 10.1038/jes.2010.32 PMC3119175 20485339

[B9] Schneider T Brouwer DH Koponen IK Jensen KA Fransmann W van Duuren-Stuurman B van Tongeren M Tielemans E Conceptual model for assessment of inhalation exposure to manufactured nanoparticles J Exp Sci and Environ Epidem 2011 1 14 10.1038/jes.2011.4 21364703

[B10] Hoppel WA Determination of the aerosol size distribution from the mobility distribution of charged fraction of aerosols Journal of Aerosol Science 1978 9 41 54 10.1016/0021-8502(78)90062-9

[B11] Wang SC Flagan R Scanning electrical mobility spectrometer Aerosol Science and Technology 1990 13 230 240 10.1080/02786829008959441

[B12] Tammet H Mirme AA Tamm E Electrical aerosol spectrometer of Tartu University J Aerosol Sci 1998 29 S1 S427 S428

[B13] Tammet H Mirme AA Tamm E Electrical aerosol spectrometer of Tartu University Atmos Res 2002 62 315 24 10.1016/S0169-8095(02)00017-0

[B14] Keskinen J Pietarinen K Lehtimäki M Electrical low pressure impactor Journal of Aerosol Science 1992 23 353 360

[B15] Baron PA Willeke K Aerosol Measurement - Principles, Techniques, and Applications 2001 New York: Wiley-Interscience ISBN 0 471 35636 0

[B16] Wilson JC Liu BY Aerodynamic particle size measurement by laser-doppler velocimetry J Aerosol Sci 1980 11 2 139 150 10.1016/0021-8502(80)90030-0

[B17] Berner A Praktische Erfahrungen mit einem 20-Stufen-Impaktor Staub - Reinhaltung der Luft 1972 32 315 320

[B18] Berner A Lürzer C Pohl F Preining O Wagner P The size distribution of the urban aerosol in Vienna The Science of the Total Environment 1979 13 245 261 10.1016/0048-9697(79)90105-0

[B19] Gorbunov B Priest ND Muir RB Jackson PR Gnewuch H A Novel Size-Selective Airborne Particle Size Fractionating Instrument for Health Risk Evaluation Ann Occup Hyg 2009 53 225 237 10.1093/annhyg/mep002 19279163 PMC2662094

[B20] Azong-Wara N Asbach C Stahlmecke B Fissan H Kaminski H Plitzko S Kuhlbusch TAJ Optimisation of a thermophoretic personal sampler for nanoparticle exposure studies J Nanoparticle Research 2009 11 1611 10.1007/s11051-009-9704-0

[B21] McMurry PH The history ofcondensation nucleus counters Aerosol Science and Technology 2000 33 297 322 10.1080/02786820050121512

[B22] Farnsworth J Caldow R A Method for Empirical Measurement of CPC Upper-end Counting Efficiency International Aerosol Conference 2010 Helsinki

[B23] Ku BK Maynard AD Comparing aerosol surface area measurement of monodisperse ultrafine silver agglomerates using mobility analysis, transmission electron microscopy and diffusion charging J Aerosol Sci 2005 36 9 1108 1124 10.1016/j.jaerosci.2004.12.003

[B24] Fissan H Neumann S Trampe A Pui DYH Shin WG Rationale and principle of an instrument measuring lungdeposited nanoparticle surface area J Nanopart Res 2007 9 53 59

[B25] Shin WG Pui DYH Fissan H Neumann S Trampe A Calibration and numerical simulation of nanoparticle surface area monitor (TSI model 3550 NSAM) J Nanopart Res 2007 9 1 61 69

[B26] Dixkens H Fissan H Development of an Electrostatic Precipitator of Off-Line Particle Analysis Aerosol Science and Technology 1999 30 438 453 10.1080/027868299304480

[B27] ISO 15900 Determination of particle size distribution - Differential electrical mobility analysis for aerosol particles 2009

[B28] Asbach C Fissan H Stahlmecke B Kuhlbusch TAJ Pui DYH Conceptual Limitations and Extensions of Lung Deposited Nanoparticle Surface Area Monitor (NSAM) J Nanopart Res 2009 11 101 109 10.1007/s11051-008-9479-8

[B29] Stahlmecke B Wagener S Asbach C Kaminski H Fissan H Kuhlbusch TAJ Investigation of airborne nanopowder agglomerate stability in an orifice under various differential pressure conditions J Nanopart Res 2009 11 7 1625 1635 10.1007/s11051-009-9731-x

[B30] Kuhlbusch TAJ Fissan H Asbach C Krug H Measurement and Detection of Nanoparticels Within the Environment Nanotechnology: Volume 2: Environmental Aspects 2008 Wiley-VCH, Weinheim 229 266

[B31] McMurry PH A review of atmospheric aerosol measurements Atmospheric Environment 2000 34 1959 1999 10.1016/S1352-2310(99)00455-0

[B32] Hinds WC Aerosol technology - properties, behaviour, and measurement of airborne particles 1999 New York: John Wiley and Sons ISBN 0 471 19410 7

[B33] Methner M Hodson L Dames A Geraci C Nanoparticle Emission Assessment Technique (NEAT) Part B J Occup and Environ Hyg 2010 7 163 176 10.1080/15459620903508066 20063229

[B34] Consent Report Tiered approach to an exposure measurement and assessment of nanoscale aerosols released from engineered nanomaterials in workplace operations, BAuA, BGRCI, IFA and VCI, Germany 2011 in press

[B35] Maynard AD Aitken RJ Assessing exposure to airborne nanomaterials: Current abilities and future requirements Nanotoxicology 2007 1 1 26 41 10.1080/17435390701314720

[B36] Brouwer D van Duuren-Stuurman B Berges M Jankowska E Bard D Mark D From workplace air measurement results towards estimates of exposure? Development of a strategy to assess exposure to manufactured nano-objects J Nanopart Res 2009 11 1867 1881 10.1007/s11051-009-9772-1

[B37] Wang YF Tsai PJ Chen CW Chen DR Hsu DJ Using a modified electrical aerosol detector to predict nanoparticle exposure to different regions of the respiratory tract for workers in Carbon Black manufacturing industry Environ Sci Technol 2010 44 6767 6774 10.1021/es1010175 20704279

[B38] Johnson DR Methner MM Kennedy AJ Steevens JA Potential for Occupational Exposure to Engineered Carbon-Based Nanomaterials in Environmental Laboratory Studies Environ Health Perspect 2010 118 1 49 54 20056572 10.1289/ehp.0901076 PMC2831966

[B39] Demou E Stark WJ Hellweg S Particle emission and exposure during nanoparticle synthesis in research laboratories Ann Occup Hyg 2009 53 829 838 10.1093/annhyg/mep061 19703918

[B40] Manodori L Benedetti A Nanoparticles monitoring in workplaces devoted to nanotechnologies J Phys Conf Ser 2009 170 012001

[B41] Park J Kwak BK Bae E Lee J Kim Y Choi K Yi J Characterization of exposure to silver nanoparticles in a manufacturing facility J Nanopart Res 2009 11 1705 1712 10.1007/s11051-009-9725-8

[B42] Bello D Wardie BL Yamamoto N deVilloria RG Garcia EJ Hart AJ Ahn K Ellenbecker MJ Hallock M Exposure to nanoscale particles and fibres during machining of hybrid advanced composites containing carbon Nanotubes J Nanopart Res 2009 5 1 231 249

[B43] Möhlmann C Welter J Klenke M Sander J Workplace exposure at nanomaterial production processes J Phys Conf Ser 2009 170 012004

[B44] Helsper C Horn HG Schneider F Wehner B Wiedensohler A Intercomparison of five mobility particle size spectrometers for measuring atmospheric submicrometer aerosol particles Gefahrst Reinhalt Luft 2008 68 475 481

[B45] Demou E Peter P Hellweg S Exposure to manufactured nanostructured particles in an industrial pilot plant Ann Occup Hyg 2008 52 8 695 706 10.1093/annhyg/men058 18931382

[B46] Yeganeh B Kull CM Hull MS Marr LC Characterization of Airborne Particles during production of carbonaceous nanomaterials Environ Sci Technol 2008 42 4600 4606 10.1021/es703043c 18605593

[B47] Peters T Elzey S Johnson R Park H Grassian V Maher T O'Shaughnessy P Airborne Monitoring to Distinguish engineered Nanomaterials from Incidental particles for environmental health and safety J Occup and Environ Hyg 2009 6 73 81 10.1080/15459620903249695 PMC4789272 19034793

[B48] Fujitani Y Kobayashi T Arashidani K Kunugita N Suemura K Measurement of physical properties of aerosols in a fullerene factory for inhalation exposure assessment J Occup and Environ Hyg 2008 5 380 389 10.1080/15459620802050053 18401789

[B49] Tsai SJ Hofmann M Hallco M Ada E Kong J Ellenbecker M Characterization and evaluation of nanoparticle release during the synthesis of single-walled and multiwalled carbon nanotubes by chemical vapor deposition Env Sci Technol 2009 43 6017 6023 10.1021/es900486y 19731712

[B50] Evans DE Ku BK Birch ME Dunn KH Aerosol monitoring during carbon nanofiber production: Mobile direct-reading sampling Ann Occup Hyg 2010 54 5 514 531 10.1093/annhyg/meq015 20447936 PMC2900095

[B51] Wang J Asbach C Fissan H Huelser T Kuhlbusch TAJ Thompson D Pui DYH How can nanobiotechnology oversight advance science and industry: Examples from environmental, health and safety studies of nanoparticles (nan-EHS) J Nanopart Res 2011 13 1373 1387 10.1007/s11051-011-0236-z

[B52] Mazzuckelli LF Methner MM Birch ME Evans DE Ku BK Crouch K Hoover MD Identification and characterization of potential sources of worker exposure to carbon nanofibres during polymer composite laboratory operations J Occup and Environ Hyg 2007 4 D125 D130 10.1080/15459620701683871 17943583

[B53] Tsai CJ Wu CH Leu ML Chen SC Huang CY Tsai PJ Ko FH Dustiness test of nanopowders using a standard rotating drum with a modified sampling train J Nanopart Res 2009 11 1 121 131 10.1007/s11051-008-9453-5

[B54] Bello D Hart AJ Ahn K Hallock M Yamamoto N Garcia EJ Ellenbecker MJ Wardle BL Particle exposure levels during CVD growth and subsequent handling of vertically-aligned carbon nanotube films Carbon 2008 46 974 981 10.1016/j.carbon.2008.03.003

[B55] Tsai SJ Ashter A Ada E Mead JL Barry CF Ellenbecker MJ Control of Airborne Nanoparticles Release During Compounding of Polymer Nanocomposites Nano 2008 3 4 301 309 10.1142/S179329200800112X

[B56] Kuhlbusch TAJ Kaminski H Jarzyna D Fissan H Asbach C Measurements of nanoscale TiO_2 _and Al_2_O_3 _in workplace environments - Methodology and results JOEH 2011 in press

[B57] Oberdörster G Toxicology of ultrafine particles. In vivo studies Phil Trans R Soc Lond A 2000 358 2719 2740 10.1098/rsta.2000.0680

[B58] Brown DM Wilson MR MacNee W Stone V Donaldson K Size-Dependent Proinflammatory Effects of Ultrafine Polystyrene Particles: A Role for Surface Area and Oxidative Stress in the Enhanced Activity of Ultrafines Toxicology and Applied Pharmacology 2001 175 191 199 10.1006/taap.2001.9240 11559017

[B59] Duffin R Tran CL Clouter A Brown DM MacNee W The Importance of Surface Area and Specific Reactivity in the Acute Pulmonary Inflammatory Response to Particles Annals of Occupational Hygiene 2002 46 Suppl 1 242 245

[B60] Asbach C Kaminski H Fissan H Monz C Dahmann D Mülhopt S Paur HR Kiesling HJ Herrmann F Voetz M Kuhlbusch TAJ Comparison of four mobility particle sizers with different time resolution for stationary exposure measurements J Nanopart Res 2009 11 7 1593 1609 10.1007/s11051-009-9679-x

[B61] Tsai SJ Ashter A Ada E Mead JL Barry CF Ellenbecker MJ Airborne nanoparticle release associated with the compounding of nanocomposites using nanoalumina as fillers Aerosol Air Qual Res 2008 8 160 177

[B62] Lee JH Kwon M Ji JH Kang CS Ahn KH Han JH Yu IJ Exposure assessment of workplaces manufacturing nanosized TiO_2 _and silver Inhal Toxicol 2011 23 4 226 236 10.3109/08958378.2011.562567 21456955

[B63] Guiot A Golanski L Tardif F Measurement of nanoparticle removal by abrasion J Phys: Conf Ser 2009 170 012014

[B64] Vorbau M Hillemann L Stintz M Method for the characterization of the abrasion induced nanoparticles release into air from surface coatings J Aerosol Sci 2009 40 3 209 217 10.1016/j.jaerosci.2008.10.006

[B65] Schneider T Jensen KA Combined Single-Drop and Rotating Drum Dustiness Test of Fine to Nanosize Powders Using a Small Drum Ann Occup Hyg 2008 52 1 23 34 18056087 10.1093/annhyg/mem059

[B66] Jensen KA Koponen IK Clausen P Schneider T Dustiness behaviour of loose and compacted Bentonite and organoclay powders: What is the difference in exposure risk? J Nanopart Res 2009 11 1 133 146 10.1007/s11051-008-9420-1

[B67] Göhler D Stintz M Vorbau M Hillemann L Characterization of nanoparticle release from surface coatings by the simulation of a sanding process Ann Occup Hyg 2010 54 6 615 624 10.1093/annhyg/meq053 20696941 PMC2918492

[B68] *ISO/DIS 12025* Nanomaterials - Quantification of nano-object release from powders by generation of aerosols 2010

[B69] Bello D Wardle BL Zhang J Yamamoto N Santeufemio C Hallock M Virji MA Characterization of exposures to nanoscale particles and fibers during solid core drilling of hybrid CNT advanced composites Int J Occup Environ Health 2010 16 434 450 21222387 10.1179/107735210799159996

[B70] Isbaseta N Biscans B Ultrafine Aerosol Emission from Free Fall of TiO2 and SiO2 Nanopowders Kona 2007 25 190 204

[B71] Maynard AD Experimental Determination of Ultrafine TiO_2 _Deagglomeration in a Surrogate Pulmonary Surfactant: Preliminary Results Ann Occup Hyg 2002 46 S1 197 20212074029

[B72] Baron PA Maynard AD Foley M Evaluation of Aerosol Release during the Handling of Unrefined Single Walled Carbon Nanotube Material 2003 NIOSH DART-02-191 10.1080/15287390490253688 14668113

[B73] Ogura I Sakurai H Gamo M Dustiness testing of engineered nanomaterials J Phys: Conf Ser 2009 170 012003

[B74] Lee SB Lee JH Bae GN Size response of an SMPS-APS system to commercial multi-walled carbon nanotubes J Nanopart Res 2010 12 2 501 512 10.1007/s11051-009-9745-4

[B75] Plitzko S Gierke E Dziurowitz N Broßell D Generation of CNT/CNF dusts by a shaker aerosol generator in combination with a thermal precipitator as the collection system for characterization of the fibre morphology Gefahrstoffe - Reinhalt Luft 2010 70 1-2 31 35

[B76] Boundy M Leith D Polton T Method to Evaluate the Dustiness of Pharmaceutical Powders Ann Occup Hyg 2006 50 5 453 458 10.1093/annhyg/mel004 16484334

[B77] Hagendorfer H Lorenz C Kaegi R Sinnet B Gehrig R Goetz NV Scheringer M Ludwig C Ulrich A Size-fractionated characterization and quantification of nanoparticle release rates from a consumer spray product containing engineered nanoparticles J Nanopart Res 2010 12 7 2481 2494 10.1007/s11051-009-9816-6

[B78] DIN 68861-2 Furniture surfaces - Behaviour at abrasion 1981

[B79] DIN EN 13523-16 Coil coated metals - Test methods - Part 16: Resistance to abrasion 2005

[B80] DIN EN ISO 7784 Paints and varnishes - Determination of resistance to abrasion 2006

[B81] Hsu L Chein H Evaluation of nanoparticle emission for TiO2 nanopowder coating materials J Nanopart Res 2007 9 1 157 163

[B82] Wohlleben W Brill S Meier MW Mertler M Cox G Hirth S von Vacano B Strauss V Treumann S Wiench K Ma-Hock L Landsiedel R On the Lifecycle of nanocomposites: Comparing Released Fragments and their In-Vivo Hazards from Three Release Mechanisms and Four Nanocomposites Small 2011 10.1002/smll.201002054 21671434

[B83] Shi T Schins RPF Knaapen AM Kuhlbusch TAJ Pitz M Heinrich J Borm PJA Hydroxyl radical generation by electron paramagnetic resonance as a new method to monitor ambient particulate matter composition J Environ Monit 2003 5 550 556 10.1039/b303928p 12948226

[B84] ISO/TS 27687 Nanotechnologies - Terminology and definitions for nano-objects - Nanoparticle, nanofibre and nanoplate 2008

